# Children and adolescents in the CoVid-19 pandemic: Schools and daycare centers are to be opened again without restrictions. The protection of teachers, educators, carers and parents and the general hygiene rules do not conflict with this

**DOI:** 10.3205/dgkh000346

**Published:** 2020-05-28

**Authors:** Peter Walger, Ulrich Heininger, Markus Knuf, Martin Exner, Walter Popp, Thomas Fischbach, Stefan Trapp, Johannes Hübner, Caroline Herr, Arne Simon, German Society for Hospital Hygiene (DGKH), German Society for Pediatric Infectious Diseases (DGPI), German Academy for Pediatric and Adolescent Medicine (DAKJ), Society of Hygiene, Environmental and Public Health Sciences (GHUP), Professional Association of Pediatricians in Germany (bvkj e.V.)

**Affiliations:** 1 German Society for Hospital Hygiene (DGKH), Berlin, Germany; 2 German Academy for Pediatric and Adolescent Medicine (DAKJ), Berlin, Germany; 3 Professional Association of Pediatricians in Germany (bvkj e.V.), Cologne, Germany; 4 German Society for Pediatric Infectious Diseases (DGPI), Berlin, Germany; 5 Society of Hygiene, Environmental and Public Health Sciences (GHUP), Munich, Germany

**Keywords:** SARS-CoV-2, children, adolescents, school, kindergarten

## Abstract

In the opinion of the medical societies of hygiene and pediatrics undersigning the present statement, the analyses published to date regarding transmission of SARS-CoV-2 and the course of CoVid-19 show that children play a much less significant role in the spread of the virus than do adults.

According to the findings available to date, not only do children and adolescents less frequently fall ill with CoVid-19, they also generally become less severely ill than do adults. The vast majority of infections in children and adolescents are asymptomatic or oligosymptomatic. Even the first analyses from China demonstrated that children and adolescents play a subordinate role in the transmission of the virus – not only to other children and adolescents, but also to adults.

Taking into account regional infection rates and available resources, daycare centers, kindergartens and elementary schools promptly should be reopened. For children, this should be possible without excessive restrictions, such as clustering into very small groups, implementation of barrier precautions, maintaining appropriate distance from others or wearing masks. A factor more decisive than individual group size is the issue of sustaining the constancy of respective group members and the avoidance of intermixing. Children can be taught basic rules of hygiene such as handwashing and careful hygiene behavior when coming into contact with others during mealtimes and/or when using sanitary facilities. Independent of the prevention measures implemented for children and adolescents, the protection of teachers, educators and caregivers is crucial, (e.g., the maintenance of appropriate distance from others, use of medical masks, situation-dependent hand disinfection, when necessary, supported by regular pool testing). Children over the age of 10 and adolescents up to school graduation age are more capable of actively understanding and conforming to specific hygiene rules. For this group, maintaining appropriate distance from others (1.5 meters), wearing a mouth-and-nose protection (whenever they are not sitting in their assigned classroom seats) and consistent education regarding the basic rules of infection prevention may provide increased options for normalizing teaching activities. Children and adolescents suspected of infection with SARS-CoV-2 should be tested immediately in order to either confirm or rule out such an infection. Evidence of individual infections in children or students must not automatically lead to the closure of the entire daycare center or school. A detailed analysis of the chain of infection is a prerequisite for a balanced approach to infection control. The opening of schools and children’s facilities should be accompanied by specifically structured, model surveillance studies that further clarify outstanding questions about infectious disease events and hygiene control. These prospective, concomitant examinations will be essential for the purpose of evaluating and verifying the effectiveness of the required hygiene measures.

## Zusammenfassung

Nach Ansicht der unterzeichnenden Hygiene- und Pädiatrie-Fachgesellschaften zeigen die bislang veröffentlichten Analysen über die Ausbreitungsdynamik von SARS-CoV-2 und die Verläufe von CoVid-19, dass Kinder im Vergleich zu Erwachsenen eine deutlich untergeordnetere Rolle in der Verbreitung des Virus einnehmen.

Kinder und Jugendliche erkranken nach den bislang vorliegenden Erkenntnissen nicht nur seltener, sondern auch im Falle einer Infektion in der Regel weniger schwer als Erwachsene. Die übergroße Mehrzahl der Infektionen im Kindes- und Jugendalter verläuft asymptomatisch oder oligosymptomatisch. Zusätzlich belegen bereits die ersten Analysen aus China, dass Kinder und Jugendliche bei der Virusübertragung auf andere Kinder und Jugendliche, aber auch auf Erwachsene eine untergeordnete Rolle spielen.

Kitas, Kindergärten und Grundschulen sollen zeitnah – unter Berücksichtigung der regionalen Neuinfektionsrate und der vorhandenen Kapazitäten – wieder eröffnet werden. Dies ist auf Seiten der Kinder ohne massive Einschränkungen, zu denen z.B. Kleinstgruppenbildung und Barriereschutzmaßnahmen wie Abstandswahrung und Maskentragen gehören würden, möglich. Entscheidender als die individuelle Gruppengröße ist die Frage der nachhaltigen Konstanz der jeweiligen Gruppe und Vermeidung von Durchmischungen. Kinder können in Grundregeln der Hygiene wie Händewaschen und achtsames Hygieneverhalten im Umgang miteinander, beim Essen und in den Sanitäreinrichtungen spielerisch und kindgerecht unterwiesen werden. Unabhängig von den bei Kindern und Jugendlichen umgesetzten Präventionsmaßnahmen ist der Schutz des Lehr-, Erziehungs- und Betreuungspersonals ganz entscheidend (Abstandswahrung untereinander, Mund-Nasen-Schutz, situationsabhängige Möglichkeit zur Händedesinfektion, ggfls. unterstützt durch regelmäßige Pooltestung). Kinder im Alter über 10 Jahre und Jugendliche bis zum Schulabschluss können aktiver in konkrete Hygieneregeln einbezogen werden. Hier erlauben eine weitgehende Abstandswahrung (1,5 m), das Tragen einer Mund-Nasen-Bedeckung (solange die Schüler nicht an dem ihnen zugewiesenen Platz sitzen) und die konsequente Erziehung in den Grundregeln der Infektionsprävention größere Spielräume für eine Normalisierung des Unterrichtsbetriebes. Kinder und Jugendliche mit V.a. eine SARS-CoV-2-Infektion sollen unverzüglich untersucht werden, um eine solche Infektion zu sichern oder auszuschließen. Der Nachweis einzelner Infektionen bei Kindern oder Schülern darf nicht automatisch zur erneuten Schließung der gesamten Kita oder Schule führen. Eine detaillierte Analyse der Infektionskette ist Voraussetzung für ein abgewogenes Infektionsmanagement. Die Öffnung der Schulen und Kindereinrichtungen sollte durch strukturierte wissenschaftliche Surveillance-Untersuchungen exemplarisch begleitet werden, die die noch offenen Fragen zum infektiologischen Geschehen und Hygienemanagement weitergehend abklären. Diese prospektiven und begleitenden Untersuchungen sind essentiell, um die Wirksamkeit der bereits jetzt geforderten Hygienemaßnahmen zu evaluieren und zu verifizieren.

## Recommendation

In the context of currently available data, we, the undersigned medical societies, consider the following to be feasible:

- Taking into account regional infection rates and available resources, daycare centers, kindergartens and elementary schools promptly should be reopened. For children, this should be possible without excessive restrictions, such as clustering into very small groups, implementation of barrier precautions, maintaining appropriate distance from others or wearing masks. A factor more decisive than individual group size is the issue of sustaining the constancy of respective group members and the avoidance of intermixing.
- Children can be taught **basic rules of hygiene such as handwashing and careful hygiene behavior** when coming into contact with others during mealtimes and/or when using sanitary facilities. This can be done in a playful and age-appropriate way. Based upon current knowledge, the implementation of such instruction, together with the mandatory equipment of all school bathrooms and handwashing sites with sufficient soap dispensers and paper towels would have considerable, positive, long-term effects on the spread of many different contagious pathogens in these facilities.
- Independent of the prevention measures implemented for children and adolescents, the protection of teachers, educators and caregivers is crucial, (e.g., the maintenance of appropriate distance from others, use of medical masks, situation-dependent hand disinfection, when necessary, supported by regular pool testing).
- If adults with a significantly elevated risk of a complicated course of SARS-CoV-2 infection live in the same household with school-age children, then individualized, creative solutions should be pursued. These should be developed following close medical consultation and with the understanding that they are a matter of personal responsibility. Their aim should be to allow children to visit community facilities. Accordingly, appropriate education and public relations work will be necessary.
- Recommendations for contact reduction via regulating size of group assembly, avoiding larger group formations during school recess breaks, during school pick-up and drop-off times and in other situations also should take into consideration home settings and extracurricular areas.
- Children over the age of 10 and adolescents up to school graduation age are more capable of actively understanding and conforming to specific hygiene rules. For this group, maintaining appropriate distance from others (1.5 meters), wearing a mouth-and-nose protection (whenever they are not sitting in their assigned classroom seats) and consistent education regarding the basic rules of infection prevention may provide increased options for normalizing teaching activities.
- In contrast to homes for the elderly, community facilities for children and adolescents do not represent a high-risk environment per se. Therefore, pursuant to individual medical considerations, these facilities also may be visited by children and adolescents with certain underlying diseases.
- Children and adolescents suspected of infection with SARS-CoV-2 should be tested immediately in order to either confirm or rule out such an infection. Evidence of individual infections in children or students must not automatically lead to the closure of the entire daycare center or school. A detailed analysis of the chain of infection is a prerequisite for a balanced approach to infection control.
- The opening of schools and children’s facilities should be accompanied by specifically structured, model surveillance studies that further clarify outstanding questions about infectious disease events and hygiene control. These prospective, concomitant examinations will be essential for the purpose of evaluating and verifying the effectiveness of the required hygiene measures.

The recommendations published here are based upon present knowledge and the interpretation thereof by the participating professional societies as of **May 19, 2020**. Although a basic matter, it nevertheless should be emphasized that advances in knowledge may lead us to reassess the situation in the coming weeks or months. Correspondingly, this may require us to adjust present recommendations.

## Background information

### Among children, infection rates and severity of SARS-CoV-2 infection are low

Current data indicate a lower rate of symptomatic infections among children and adolescents than among adults. The majority of children and adolescents with SARS-CoV-2 infection show either no symptoms or else only mild symptoms [1], [2], [3]. Severe courses of the disease rarely occur [4]. Although not exclusively, only half of all serious infections affect children with underlying diseases and/or with treatment-related impairments to the immune system [5]. Hospital admission is not always an indicator of disease severity. As Parri et al. report (Coronavirus Infection in Pediatric Emergency Departments Study, 17 emergency departments in Italy), of 100 children, 38 were admitted to hospital, but only one of the children was measured as having a pulse oximetric oxygen saturation below 92% [6].

The registry of the German Society for Pediatric Infectious Diseases (DGPI) (updated as of May 18, 2020; n=138) reports that among children treated on an inpatient basis, 24% of those on regular hospital wards and 56% of those on intensive care units had a relevant underlying disease. (The ICU patients represented 15% of all inpatient children; n=20.)

Deaths among children and adolescents are extremely rare. (As of May 18, 2020, there has been just one CoViD-19-associated death documented in the DGPI’s registry of inpatient pediatric cases.) In other countries too, to date there have been only a few isolated fatalities. (Concerning the SARS-CoV-2-associated multisystemic hyperinflammatory syndrome, please see further information below.)

A pre-existing, significantly increased susceptibility to severe respiratory tract infections, (e.g., from influenza or other respiratory viruses such as respiratory syncytial virus (RSV), human metapneumovirus (hMPV) or other human pathogenic coronaviruses), is already known to parents as well as treating physicians – and this is independent of the current CoVid-19 pandemic.

To what extent specific medical history and/or underlying disease increase the risk of a complicated course in childhood infections with SARS-CoV-2 is not conclusively known. As an example, pediatric oncologists from the MSK Kids Pediatric Program of the Memorial Sloan Kettering Cancer Center (New York) found no increased rate of complications among 20 pediatric oncology patients who tested positive in the middle of the outbreak there [7]. This confirms the very early results of an international survey published on April 20, 2020 [8]. In a recent report from the pediatric oncology department in Padua, Italy, (a high-prevalence region for COVID-19), not a single SARS-CoV-2 case was found in over 500 individual tests, including both patients and accompanying persons [9].

Among all those who tested positive, the percentage of children under 10 years of age so far is between 1 and 2%. Positive rates reach a maximum of 6% by the age of 20 years. In Germany, the proportion of children <10 years was 1.9% and those between 10 and 19 years old was 4.3%. According to the RKI’s status report from May 17, 2020 [10], a total of 174,355 infections were recorded by that date. Of these, 3,295 were under 10 years old and 7,524 were between 10 and 19 years old. Only three deaths had occurred among those between three and 18 years old, and each of these cases had underlying conditions.

In Norway, as of March 22, 2020, the percentage of SARS-CoV-2-positive children and adolescents up to the age of 20 was 4%. Due to a high proportion of asymptomatic children with SARS-CoV-2 infection, it may be assumed that this represents an underreporting reporting of cases. **The actual infection rate cannot be known beyond doubt. Available results from seroepidemiological studies are not yet sufficient for the purpose of determining the actual infection prevalence among children and adolescents.** To pediatricians, it will not be surprising to hear that even minimally symptomatic children can excrete the virus in nasopharyngeal secretions in the same concentration as symptomatic adults [11], [12]. However, to deduce from this that there may be a higher risk of transmission from children to other persons (and especially to adults) [11], stands in contradiction to the observation that in most confirmed pediatric SARS-CoV-2 cases, it was an adult contact person (e.g., a parent) who was the original source of infection. Of greater relevance is the fact that in contrast to adults, children do not appear to have elevated virus concentrations in the upper respiratory tract [11], [12].

### Risk of transmission by children seems low – school and daycare center closures are likely to have only a narrow impact on the further spread of infection

Numerous findings speak against an increased risk of infection from children. Various investigations, reviews, outbreak and cluster analyses, models analyzing previous influenza pandemics (see below), as well as analyses published in relation to the earlier MERS and SARS-1 coronavirus pandemics, are painting an increasingly convincing picture that, in contrast to their part in influenza transmission, children do not play a prominent role in the disease transmission dynamics of the current CoVid-19 pandemic.

Within families, transmission of infection to children usually originates from infected adults [13], whereas evidence of transmission from an infected child to adults remains lacking. Such transmission events also may exist, but they appear to be of less immediate consequence. The impact of school and daycare center closures on the dynamics of further infection spread of SARS-CoV-2 is estimated to be limited [14], [15]. Key data used as evidence for the significant role of children in transmission dynamics have been taken from studies of influenza pandemics. Comparable data from coronavirus pandemics do not exist. This further demonstrates the relatively low importance of transmission by children.

- **WHO-China Joint Mission Report:** The report summarizes data on the risk of infection for children <18 years in China: There is a low infection rate in China. (Children account for 2.4% of all confirmed infections). In Wuhan, from November/December 2019 through January 15, 2020, subsequent tests of secretions of children with respiratory symptoms with influenza-like illness did not detect SARS-CoV-2. Through intensive contact tracing, CoVid-19 was detected in approximately 1-5% of contact persons. Infected children largely were found to live in households that also had infected adults. The infection rate of children in these households was between 3–10%. The Joint Mission team found no evidence of transmission from infected children to adults [16].
- **Germany:** In the current DGPI registry, where cases of children hospitalized with SARS-CoV-2 are being recorded, 81% of children with a reconstructed chain of infection became infected by their parents [5]. During the period March 18–May 18, 2020, the DGPI registry logged just 138 inpatient admissions of children with SARS-CoV-2 in Germany. (87% of these children already had been discharged, with 75% fully recovered and 25% having minor residual symptoms.) Even in areas deemed hotspots – ones where there were high numbers of adult patients who had been ventilated – there were only isolated cases of pediatric patients in pediatric clinics, according to the South German Society for Pediatric and Adolescent Medicine (SGKJ). Specifically, cases appeared in the following locations: Rosenheim (hotspot Rosenheim district), 1 child with SARS-CoV-2; Passau (hotspot Rottal-Inn district), no children hospitalized with SARS-CoV-2; city of Baden-Baden, 1 child hospitalized with SARS-CoV-2; city of Munich (von Hauner’sches Children’s Hospital), 2 children hospitalized with SARS-CoV-2 to date.
- **France:** One analysis describes a hyper-spreader event in the French Alps, whereby 11 of 16 hotel guests were infected with SARS-CoV-2 by an asymptomatic SARS-CoV-2-positive tourist (index patient) (12/16=75% infection rate). Among the hotel guests was a 9-year-old child with mild symptoms of respiratory infection and a co-infection with influenza and picorna viruses. For one week, this patient attended three schools in addition to a skiing school. In relation to this case, 172 contacts were traced and tested, including 73 with virus swabs. Of the contacts, 112 were from the child’s school, (84 with moderate risk of transmission and 88 with low risk of transmission, as measured by the length of contact). Among these, 70 had respiratory symptoms and 46 had other respiratory viruses, (33% influenza, 18% picorna viruses, 16% classic coronaviruses). Not a single SARS-CoV-2 infection was found among the child’s contacts. The authors conclude: *"The fact that an infected child did not transmit the disease despite close interactions within schools suggests potential different transmission dynamics in children."* [17] In a major outbreak at a secondary school during the early phase of the epidemic in France, serological testing for SARS-CoV-2 was carried out approximately eight weeks after the start of the infection spread: 40% of 15- to 17-year-olds and 43 percent of teachers tested positive, but only 2.7% of those under 15 years old tested positive. The transmission rate within families was 11.4% for parents and 10.2% for siblings [18]. Salje et al. assume that by May 11, 2020, 4.4% (2.8–7.2%) of the French population had been infected with SARS-CoV-2. This would correspond to a total of 2.8 million people, (1.8–4.7; calculated hospitalization rate across all age groups 3.6%, mortality 0.7%, under-20s 0.001%) [19].
- **Iceland:** In the context of a study, 6% of the Icelandic population was tested between January 31 and early April 2020 [20]. Three groups were examined: 9,199 persons at high risk (presence of symptoms, positive travel history after a skiing holiday in Austria or Northern Italy, and/or contact with a high-risk person). In this group, the total infection rate was 13.3%, with infection among children <10 years at 6.7%. Following an open invitation to participate in testing (population sample), an additional 10,797 persons were tested by March 13, 2020. In this group, the total infection rate was 0.8%, with infection among children <10 years at 0%. Following a targeted invitation (representative sample for quality comparison purposes), 2,283 persons were additionally tested by April 4, 2020. In this group, the total infection rate was 0.6%. At the beginning of the pandemic’s spread in Iceland, travel history (return from ski resorts in Austria and northern Italy) represented the main risk factor. Later, travel history (return from Great Britain) and intra-family transmission were the predominant factors [20]. Children <10 years played almost no role in the transmission dynamics. These data have been further confirmed as part of follow-up testing, during which the overall test rate rose to >15% of the total population.
- In an analysis by Swiss scientists regarding current effects of non-pharmaceutical interventions (NPIs) on the number of infections in 20 countries (USA, EU-15, Norway, Switzerland, Canada and Australia) [21], it was shown that school closures had the second-lowest effect on infection transmission (11%). According to the authors, this finding is consistent with previous literature showing that transmission of SARS-CoV-2 by children has been comparatively low. In this analysis, the closing of event venues (33%) and of businesses belonging to non-critical infrastructure (28%) have had a stronger impact. The factor with the very lowest effect was the generalized contact ban [21].
- **Great Britain:** Published on April 6, 2020, a systematic review [15] assessed the results of 16 studies that had examined the impact of school closures on coronavirus pandemics. In the SARS-1 outbreaks in China, Hong Kong and Singapore in 2003, school closures showed limited benefit in slowing the spread of the virus. The authors point to a variety of collateral damage (loss of essential labor due to the need for parents to provide childcare; restrictions on learning, sociability and physical activity opportunities for students; and significant psychosocial risks for children who are most vulnerable, including those from low-income families). In the absence of solid data on current effects of the CoVid-19 pandemic, the authors conduct an in-depth examination of findings from influenza pandemics. In contrast to these pandemics, they conclude that the effect of school closures on the CoVid-19 pandemic is likely to be small. Interactions with other factors, (e.g., moment and timing of school closure, parents working from home, additional social mixing, including close contacts with at-risk individuals in the family), must be taken into account. The authors stress the urgent need for meaningful data and describe these findings as a dilemma for policymakers, because decisions about school and daycare center closures are getting made without reliable evidence regarding the effectiveness of such measures. In another model analysis conducted by the CoVid-19 Response Team at Imperial College in London – one conducted in order to evaluate non-pharmaceutical interventions (NPIs) – the authors conclude that school closures only can be understood to have an effect if or when it is assumed that children will play a significant role in transmission dynamics. Their model calculates that school closures have only a very minimal effect on the mortality rate [14].
- With respect to the current CoVid-19 pandemic, the relevance of school closures to transmission dynamics is hereby negated. This is in distinct contrast to the role that such closures may play during influenza pandemics. The longer school closures last, the more considerable the collateral damage they will cause. This must be taken into account [22], [23], [24], [25], [26].
- **Norway:** In a systematic literature analysis commissioned by the Norwegian Institute of Public Health (NIPH), the authors summarize their conclusions as follows: Children appear to be less susceptible to a symptomatic infection with SARS-CoV-2 than are adults. The authors evaluate the current literature, especially with respect to cluster analyses of family transmissions in China, and conclude that, based upon current data, children do not play a significant role as vectors of virus transmission. However, it may be too early to pass final judgment (as of March 2020). On the question of the effectiveness of school and daycare center closures, the authors emphasize the lack of data on coronavirus epidemics and explain that current experience and analyses refer almost exclusively to influenza epidemics where the role of children is known to be more important with respect to transmission. For this reason, relevant disease knowledge is not easily transferable from one context to the other: *“We have not found any research reports that have calculated the effects of school/kindergarten closures during the COVID-19 epidemic. There are a number of systematic reviews on this issue, but they are mostly based only on studies done in connection with influenza epidemics. It is highly uncertain how relevant the experiences from influenza epidemics are in connection with the COVID-19 epidemic, as it is quite possible that children play a small role in the transmission of the SARS-CoV-2, as opposed to what is the case with the influenza virus.”* [3]
- **Netherlands:** In the Netherlands, all municipal health services (GGDs) conduct contact tracing via in-depth analyses of infection chains. According to these analyses, no patients under 18 years of age were found to have infected other people. Approximately 40 general practitioners in the Netherlands registered the number of their patients with flu-like symptoms who visited their practices. A total of 6.5% of patients was shown to be infected with SARS-CoV-2. No SARS-CoV-2 infection was found among those tested who were under 20 years old. In the context of a long-term seroepidemiological study (PIENTER Corona Study), 2,096 people were examined as of April 17, 2020. The first results show that 3.6% of those examined had SARS-CoV-2 antibodies in their blood. Of these, 2% were under 20 years old and 4.2% were adults. Dutch health authorities interpret this data as evidence that children play only a minor role in the transmission of CoVid-19 [27].
- In a report by the **National Centre for Immunisation Research and Surveillance** (NCIRS, Australia, April 26, 2020) which tracked on SARS-CoV-2 infections among students (n=9) and teachers (n=9) from 15 schools in New South Wales (10 secondary schools and 5 primary schools), subsequent transmission to classmates (n=735) and staff (n=128) was investigated. During the school day, index patients had normal contact with others. At the primary schools, 137 students and 31 staff were classified as “close contact persons” (those who had face-to-face contact for 15 minutes and/or who were in the same room for at least two hours). At the secondary schools, there were 598 students and 97 staff. No subsequent SARS-CoV-2 infection became detected in any of the staff examined (teachers, caregivers, etc.). (30% were tested.) Only two other children, (a primary school child and a secondary school student), may have been infected by one of the index patients [28].
- **Virus concentrations in the throat and suspected contagion:** In a quantitative analysis of the viral load among CoVid-19 patients, no significant age-related differences were found. Based upon nasopharyngeal virus identification from tests following clinical indication, the authors conclude: *“… In particular, these data indicate that viral loads in the very young do not differ significantly from those of adults. Based on these results, we have to caution against an unlimited re-opening of schools and kindergartens in the present situation. Children may be as infectious as adults.”* However, given that investigations so far predominantly have focused upon symptomatic children and that the overall number of cases has been small, the admissibility of determining transmission risk based upon quantitative viral load in the upper respiratory tract seems questionable. It also may strike some as unusual that, despite the obvious need for discussion, the warning regarding an “unlimited” reopening of kindergartens and schools already has been emphasized in the article’s abstract – even though the epidemiological data and comparisons with analyses of previous coronavirus and influenza pandemics is considered controversial [11]. Interestingly, in an investigation of 23 symptomatic newborns, children and adolescents, L’Huillier et al. (Geneva) come to the same conclusion with respect to quantitative virus detection [12]. Nevertheless, they determine only that transmission through children is possible in principle.
- In a systematic **analysis of confirmed COVID-19 cases reported to the RKI during the lockdown** (since March 16, 2020), Goldstein et al. found there to be a relative increase in prevalence among 15- to 20-year-old adolescents/young adults, as compared to patients who were over 25 years old. The group with the highest relative increase in prevalence was that of 20- to 25-year-olds [29]. The authors conclude that 15- to 25-year-olds may be playing an important role in the spread of SARS-CoV-2 infection. However, according to this analysis (during the lockdown), this definitely was not applicable to children under 15 years old [29]. Following in-depth mathematical modelling, the same working group concluded that it may be necessary to maintain social distancing measures until 2022 [30]. In an editorial from The Lancet Child & Adolescent Health [26], the authors describe the relevance of characteristics particular to adolescence, an age during which insecure tendencies, along with newfound explorations and rebellion against social norms, are part of normal development. These developmental factors should be taken into account during the intermediation and review of prevention measures.

### Multisystemic hyperinflammatory syndrome in children following SARS-CoV-2 infection 

This very rare syndrome, which to date only has been provisionally clinically defined [31], [32], is temporally associated with SARS-CoV-2 infection in children and appears similar to other childhood hyperinflammatory syndromes, (e.g., Kawasaki Syndrome, Macrophage Activation Syndrome). The syndrome can start with severe gastrointestinal symptoms and become life-threatening if the coronary arteries become affected, (see Kawasaki syndrome) [33], [34], [35].

**On May 6, 2020, the DGPI, together with the German Society for Pediatric Cardiology, published a first statement on this subject** [36]. The Royal College of Paediatrics and Child Health also has published initial guidance on the syndrome’s diagnosis and therapy [32]. Closely following the publication of such communications is important for all physicians who are currently treating children and adolescents. This applies to both outpatient and inpatient treatment contexts. The pathogenetic relationship with a previous SARS-CoV-2 infection remains unclear [37]. Suspected cases should be monitored and treated in hospital at an early stage and also reported to the relevant health authorities. **The occurrence of this multisystemic hyperinflammatory syndrome remains so rare vis-à-vis the total number of children infected with SARS-CoV-2 that it does not alter the basic conclusions we present here.**

### Conclusions

In the opinion of the medical societies undersigning the present statement, the analyses published to date regarding transmission of SARS-CoV-2 and the course of CoVid-19 show that children play a much less significant role in the spread of the virus than do adults.

This conclusion in no way negates the need for carefully conducted, prospective surveillance, supported and accompanied by broad-scale test indications, when schools and daycare centers reopen.

However, the present statement should provide key guidance for related socio-political decisions undertaken in the context of pandemic management. According to the findings available to date, not only do children and adolescents less frequently fall ill with CoVid-19, they also generally become less severely ill than do adults. The vast majority of infections in children and adolescents are asymptomatic or oligosymptomatic. Even the first analyses from China demonstrated that children and adolescents play a subordinate role in the transmission of the virus – not only to other children and adolescents, but also to adults.

Although it is possible that the risk of transmission from adolescents over 15 years old does not significantly differ from that from adults, aspects relating to compliance with prevention measures nevertheless may play an important role. Especially in children under 10 years of age, current data suggest both a lower rate of infection and a significantly lower rate of contagion. Currently, there is insufficient evidence to explain the cause of this lower virus transmission rate, which should be addressed by follow-up investigations.

Even among symptomatic children infected by other respiratory viruses, isolated case evidence shows there to be no significant transmission of SARS-CoV-2 [18]. Because of this, the lower risk of transmission may be related either to the fact that children cough less or that the duration of symptoms is shorter. There is no linear relationship between the viral load detected in the upper respiratory tract and the risk of transmission, since it is the quantity of virus reaching the recipient’s mucous membranes that ultimately determines whether or not an infection takes hold.

The mounting evidence provided by this data has prompted British scientists to call for schools to be reopened immediately, including for children with pre-existing underlying conditions [24]. In the opinion of these British scientists, governments worldwide should allow all children to return to school, regardless of comorbidity.

Although current analyses may provide explanation for why school closures are ineffective in the context of the current CoVid-19 pandemic, detailed monitoring nevertheless will be needed in order to confirm the safety of this approach [21], [15]. According to the current state of knowledge in Germany, severe CoViD-19 is by no means more common than many other, potentially severe infectious diseases in children – ones that to date have not led to the closure of schools or other children’s facilities.

Doctors should provide individualized risk assessments and customize decision-making for those at particular risk, such as children who are in their first months following bone marrow or organ transplantation, or those who suffer from severe congenital immune deficiencies.

If adults with a significantly elevated risk of a complicated course of SARS-CoV-2 infection live in the same household with school-age children, then individualized, creative solutions should be pursued. These should be developed following close medical consultation and with the understanding that they are a matter of personal responsibility. Their aim should be to allow children to visit community facilities.

Upon evaluating the available data regarding the non-medical consequences of closing community facilities, as well as regarding the infectious disease characteristics of SARS-CoV-2 and the epidemiological situation in Germany, on April 20, 2020, the DAKJ began recommending “... the resumption of school attendance ... for all children and adolescents at the earliest possible date.”

Here, the DAKJ additionally points out that before or during the closure of community facilities for children and adolescents, the consequences of this action for this group had not been thoroughly discussed. The concerns of those directly affected, along with their advocates, had not been sufficiently heard, an issue which represented a disregard for the fundamental rights of children [22], [23], [38].

## Notes

### Acknowledgement

The authors, who have coordinated consensus finding, would like to express their sincere thanks to the numerous dedicated colleagues from the medical associations who actively contributed to developing the consensus for this statement. 

They also thank Natalie Diffloth for editing the English version.

Prof. Dr. med. Ulrich Heininger is speaking for the members of the DAKJ’s Infectious Diseases and Vaccination Issues Commission: Dr. med. Herbert Grundhewer, Prof. Dr. med. Ulrich Heininger, Prof. Dr. med. Markus Knuf, Prof. Dr. med. Georg-Christoph Korenke, Prof. Dr. med. Andreas Müller, Dr. med. Ulrich von Both

### Competing interests

The authors declare that they have no competing interests.

## References

[1] CDC COVID-19 Response Team (2020). Coronavirus Disease 2019 in Children – United States, February 12-April 2, 2020. MMWR Morb Mortal Wkly Rep.

[2] Chidini G, Villa C, Calderini E, Marchisio P, De Luca D (2020). SARS-CoV-2 Infection in a Pediatric Department in Milan: A Logistic Rather Than a Clinical Emergency. Pediatr Infect Dis J.

[3] Fretheim A (2020). The role of children in the transmission of SARS-CoV-2 (COVID-19) – a rapid review.

[4] Dong Y, Mo X, Hu Y, Qi X, Jiang F, Jiang Z, Tong S (2020). Epidemiology of COVID-19 Among Children in China. Pediatrics.

[5] Armann J, Simon A, Diffloth N, Doenhardt M, Hufnagel M, Trotter A, Schneider D, Hübner J, Berner R (2020). Hospital admission in children and adolescents with COVID-19 – early results from a national survey conducted by the German Society for Pediatric Infectious Diseases (DGPI). Dtsch Arztebl Int.

[6] Parri N, Lenge M, Buonsenso D, Coronavirus Infection in Pediatric Emergency Departments (CONFIDENCE) Research Group (2020). Children with Covid-19 in Pediatric Emergency Departments in Italy. N Engl J Med.

[7] Boulad F, Kamboj M, Bouvier N, Mauguen A, Kung AL (2020). COVID-19 in Children With Cancer in New York City. JAMA Oncol.

[8] Hrusak O, Kalina T, Wolf J, Balduzzi A, Provenzi M, Rizzari C, Rives S, Del Pozo Carlavilla M, Alonso MEV, Domínguez-Pinilla N, Bourquin JP, Schmiegelow K, Attarbaschi A, Grillner P, Mellgren K, van der Werff Ten Bosch J, Pieters R, Brozou T, Borkhardt A, Escherich G, Lauten M, Stanulla M, Smith O, Yeoh AEJ, Elitzur S, Vora A, Li CK, Ariffin H, Kolenova A, Dallapozza L, Farah R, Lazic J, Manabe A, Styczynski J, Kovacs G, Ottoffy G, Felice MS, Buldini B, Conter V, Stary J, Schrappe M (2020). Flash survey on severe acute respiratory syndrome coronavirus-2 infections in paediatric patients on anticancer treatment. Eur J Cancer.

[9] Sainati L, Biffi A (2020). How we deal with the COVID-19 epidemic in an Italian paediatric onco-haematology clinic located in a region with a high density of cases. Br J Haematol.

[10] Robert Koch Institut (RKI) Täglicher Lagebericht des RKI zur Coronavirus-Krankheit-2019 (COVID-19).

[11] Jones T, Mühlemann B, Veith T, Zuchowski M, Hofmann J, Stein A, Edelmann A, Corman V, Drosten C (2020). An analysis of SARS-CoV-2 viral load by patient age [Preprint]. https://virologie-ccm.charite.de/fileadmin/user_upload/microsites/m_cc05/virologie-ccm/dateien_upload/Weitere_Dateien/analysis-of-SARS-CoV-2-viral-load-by-patient-age-v2.pdf.

[12] L’Huillier A, Torriani G, Pigny F, Kaiser L, Eckerle I (2020). Shedding of infectious SARS-CoV-2 in symptomatic neonates, children and adolescents [Preprint]. medRxiv.

[13] Ghinai I, Woods S, Ritger KA, McPherson TD, Black SR, Sparrow L, Fricchione MJ, Kerins JL, Pacilli M, Ruestow PS, Arwady MA, Beavers SF, Payne DC, Kirking HL, Layden JE (2020). Community Transmission of SARS-CoV-2 at Two Family Gatherings - Chicago, Illinois, February-March 2020. MMWR Morb Mortal Wkly Rep.

[14] Ferguson N, Laydon D, Nedjati Gilani G, Imai N, Ainslie K, Baguelin M, Bhatia S, Boonyasiri A, Cucunuba Perez Z, Cuomo-Dannenburg G, Dighe A, Dorigatti I, Fu H, Gaythorpe K, Green W, Hamlet A, Hinsley W, Okell L, Van Elsland S, Thompson H, Verity R, Volz E, Wang H, Wang Y, Walker P, Walters C, Winskill P, Whittaker C, Donnelly C, Riley S, Ghani A, Imperial College COVID-19 Response Team (WHO Collaborating Centre for Infectious Disease Modelling / MRC Centre for Global Infectious Disease Analysis / Abdul Latif Jameel Institute for Disease and Emergency Analytics) (2020). Report 9 – Impact of non-pharmaceutical interventions (NPIs) to reduce COVID-19 mortality and healthcare demand.

[15] Viner RM, Russell SJ, Croker H, Packer J, Ward J, Stansfield C, Mytton O, Bonell C, Booy R (2020). School closure and management practices during coronavirus outbreaks including COVID-19: a rapid systematic review. Lancet Child Adolesc Health.

[16] WHO-China Joint Mission on Coronavirus Disease (2020). Report of the WHO-China Joint Mission on Coronavirus Disease 2019 (COVID-19).

[17] Danis K, Epaulard O, Bénet T, Gaymard A, Campoy S, Bothelo-Nevers E, Bouscambert-Duchamp M, Spaccaferri G, Ader F, Mailles A, Boudalaa Z, Tolsma V, Berra J, Vaux S, Forestier E, Landelle C, Fougere E, Thabuis A, Berthelot P, Veil R, Levy-Bruhl D, Chidiac C, Lina B, Coignard B, Saura C, Investigation Team (2020). Cluster of coronavirus disease 2019 (Covid-19) in the French Alps, 2020. Clin Infect Dis.

[18] Fontanet A, Tondeur L, Madec Y, Grant R, Besombes C, Jolly N, Fernandes Pellerin S, Ungeheuer M, Cailleau I, Kuhmel L, Temmam S, Huon C, Chen K, Crescenzo B, Munier S, Demeret C, Grzelak L, Staropoli I, Bruel T, Gallian P, Cauchemez S, van der Werf S, Schwartz O, Eloit M, Hoen B (2020). Cluster of COVID-19 in northern France: A retrospective closed cohort study [Preprint]. medRxiv.

[19] Salje H, Tran Kiem C, Lefrancq N, Courtejoie N, Bosetti P, Paireau J, Andronico A, Hozé N, Richet J, Dubost CL, Le Strat Y, Lessler J, Levy-Bruhl D, Fontanet A, Opatowski L, Boelle PY, Cauchemez S (2020). Estimating the burden of SARS-CoV-2 in France. Science.

[20] Gudbjartsson DF, Helgason A, Jonsson H, Magnusson OT, Melsted P, Norddahl GL, Saemundsdottir J, Sigurdsson A, Sulem P, Agustsdottir AB, Eiriksdottir B, Fridriksdottir R, Gardarsdottir EE, Georgsson G, Gretarsdottir OS, Gudmundsson KR, Gunnarsdottir TR, Gylfason A, Holm H, Jensson BO, Jonasdottir A, Jonsson F, Josefsdottir KS, Kristjansson T, Magnusdottir DN, le Roux L, Sigmundsdottir G, Sveinbjornsson G, Sveinsdottir KE, Sveinsdottir M, Thorarensen EA, Thorbjornsson B, Löve A, Masson G, Jonsdottir I, Möller AD, Gudnason T, Kristinsson KG, Thorsteinsdottir U, Stefansson K (2020). Spread of SARS-CoV-2 in the Icelandic Population. N Engl J Med.

[21] Banholzer N, van Weenen E, Kratzwald B, Seeliger A, Tschernutter D, Bottrighi P, Cenedese A, Puig Salles J, Vach W, Feuerriegel S (2020). Impact of non-pharmaceutical interventions on documented cases of COVID-19 [Preprint]. medRxiv.

[22] Deutsche Akademie für Kinder- und Jugendmedizin e.V. (2020). Maßnahmen zur Prävention einer SARS-CoV-2 Infektion bei Kindern mit besonderem Bedarf bei der Betreuung in Gemeinschaftseinrichtungen (GE).

[23] Deutsche Akademie für Kinder- und Jugendmedizin e.V. (2020). Stellungnahme der Deutschen Akademie für Kinder- und Jugendmedizin e.V. zu weiteren Einschränkungen der Lebensbedingungen von Kindern und Jugendlichen in der Pandemie mit dem neuen Coronavirus (SARS-CoV-2).

[24] Munro APS, Faust SN (2020). Children are not COVID-19 super spreaders: time to go back to school. Arch Dis Child.

[25] Schober T, Rack-Hoch A, Kern A, von Both U, Hübner J (2020). Coronakrise: Kinder haben das Recht auf Bildung – Als Überträger von SARS-CoV-2 spielen Kinder eine geringere Rolle als bislang vermutet. Daher sollten die Schließungen von Kindertagesstätten und Schulen neu überdacht werden. Dtsch Arztebl.

[26] The Lancet Child Adolescent Health (2020). Pandemic school closures: risks and opportunities. Lancet Child Adolesc Health.

[27] Rijksinstituut voor Volksgezondheid en Milieu (Netherlands National Institute for Public Health and the Environment) (2020). Children and COVID-19.

[28] National Centre for Immunisation Research and Surveillance (NCIRS) (2020). Report: COVID-19 in schools - the experience in NSW.

[29] Goldstein E, Lipsitch M (2020). Temporal rise in the proportion of younger adults and older adolescents among coronavirus disease (COVID-19) cases following the introduction of physical distancing measures, Germany, March to April 2020. Euro Surveill.

[30] Kissler SM, Tedijanto C, Goldstein E, Grad YH, Lipsitch M (2020). Projecting the transmission dynamics of SARS-CoV-2 through the postpandemic period. Science.

[31] Centers for Disease Control and Prevention (CDC) (2020). Multisystem Inflammatory Syndrome in Children (MIS-C) Associated with Coronavirus Disease 2019 (COVID-19).

[32] Royal College of Paediatrics and Child Health (2020). Guidance: Paediatric multisystem inflammatory syndrome temporally associated with COVID-19.

[33] European Centre for Disease Prevention and Control (ecdc) (2020). Rapid risk assessment: Paediatric inflammatory multisystem syndrome and SARS -CoV-2 infection in children.

[34] Riphagen S, Gomez X, Gonzalez-Martinez C, Wilkinson N, Theocharis P (2020). Hyperinflammatory shock in children during COVID-19 pandemic. Lancet.

[35] Verdoni L, Mazza A, Gervasoni A, Martelli L, Ruggeri M, Ciuffreda M, Bonanomi E, D’Antiga L (2020). An outbreak of severe Kawasaki-like disease at the Italian epicentre of the SARS-CoV-2 epidemic: an observational cohort study. Lancet.

[36] Deutsche Gesellschaft für Pädiatrische Infektiologie (DGPI), Deutsche Gesellschaft für Pädiatrische Kardiologie (DGPK) (2020). Gemeinsame Stellungnahme der Deutschen Gesellschaft für Pädiatrische Infektiologie (DGPI) und der Deutschen Gesellschaft für Pädiatrische Kardiologie und Angeborene Herzfehler (DGPK). Hyperinflammationssyndrom im Zusammenhang mit COVID-19 (Stand 06.05.2020).

[37] Jones VG, Mills M, Suarez D, Hogan CA, Yeh D, Bradley Segal J, Nguyen EL, Barsh GR, Maskatia S, Mathew R (2020). COVID-19 and Kawasaki Disease: Novel Virus and Novel Case. Hosp Pediatr.

[38] Deutsche Gesellschaft für Pädiatrische Infektiologie, Deutsche Akademie für Kinder- und Jugendmedizin e. V. (2020). Gemeinsame Stellungnahme von DGPI und Deutsche Akademie für Kinder- und Jugendmedizin (DAKJ) zum aktuellen Umgang mit SARS-CoV-2, dem Erreger von Coronavirus disease-19 (COVID-19). Stand 21.3.2020.

